# Defeating cancer: the 150 most important questions in cancer research and clinical oncology

**DOI:** 10.1186/s40880-016-0165-4

**Published:** 2016-12-28

**Authors:** Chao-Nan Qian, Wei Zhang, Rui-Hua Xu

**Affiliations:** 1 State Key Laboratory of Oncology in South China, Collaborative Innovation Center for Cancer Medicine, Sun Yat-sen University Cancer Center, Guangzhou, 510060 Guangdong P. R. China; 2 Wake Forest Baptist Comprehensive Cancer Center, Winston-Salem, NC 27157-1082 USA

Despite all the human efforts and monetary investment over the last few decades, cancer is still a devastating threat to our life expectancy and quality of life in many parts of the world. The etiology of cancer varies. The genetic and epigenetic causes of cancer are heterogeneous and multifaceted. Early detection is still a difficult issue in many types of malignancies, resulting in unsatisfying cure rates. Cancer not only is a disease of malignant cells but also involves the host physiology, especially the natural defensive immune systems. The task of overcoming cancer is so vast and overwhelming that it will no doubt require scientists worldwide to join efforts and resources to succeed.

The year of 2016 is the 150th year Anniversary of Dr. Sun Yat-sen’s birth. Dr. Sun was a doctor who devoted his life to treating patients and to revolutionizing the history of mankind by putting China on a path for modernization. We seek to honor Dr. Sun Yat-sen’s legacy by soliciting 150 most important questions in the war against cancer from cancer researchers and clinicians worldwide. We hope that presenting the most important questions in cancer research and clinical oncology would be beneficial to stimulating all cancer fighters, especially young generation researchers to focus on the key issues that will generate the most significant impact on cancer cures.

A significant number of questions have been submitted by clinicians and researchers covering the fields of cancer etiology, cancer immunology, metabolism, biology, prevention, early detection, treatment modality, drug development, novel experimental technology, and cancer-induced erythrogenesis.

Starting in the December issue of 2016, the *Chinese Journal of Cancer* will publish a series of peer-reviewed top-ranked questions from several categories. We hope these questions will further trigger a brainstorm among cancer researchers and encourage continued submission of key questions and research articles in the coming months to the *Chinese Journal of Cancer* as a special tribute to Dr. Sun Yat-sen.

